# The current trend of genomics research for human diseases

**DOI:** 10.1186/1755-8794-6-S1-S1

**Published:** 2013-01-23

**Authors:** Ke K Zhang, Hamid R Arabnia, Yunliang Wang, Youping Deng

**Affiliations:** 1 Department of Pathology, Bioinformatics Core, School of Medicine and Health Sciences, University of North Dakota, Grand Forks, ND 58201, USA; 2 Department of Computer Science, University of Georgia, Athens, Georgia 30602-7404, USA; 3 Department of Neurology, The 148 Hospital of PLA, Zibo, Shandong, 255300, China; 4 Department of Internal Medicine and Biochemistry, Rush University Cancer Center, Rush University Medical Center, Chicago, IL 60612, USA

## Abstract

This is an introduction to the supplement to *BMC Medical Genomics *that includes16 papers selected from the 2011 World Congress in Computer Science, Computer Engineering, Applied Computing as well as other sources with a focus on genomics studies with a focus on human diseases.

## 

This supplement is dedicated to the World Congress in Computer Science, Computer Engineering, and Applied Computing on July 18-21 in 2011 in Las Vegas, Nevada. The congress drew a wide attention from computer scientists, applied mathematicians and statisticians, bioinformaticians, and researchers in many other fields. As one of the fastest developing fields, genomics became a prominent area that drew attentions from most attendants. Noting that a substantial part of genomics work was dedicated to human disease studies, we decided to launch a supplement to *BMC Medical Genomics *with manuscripts selected from this congress as well as invited submissions. More than 30 manuscripts were submitted to this supplement. Each manuscript was reviewed by at least two reviewers in addition to the review by the editorial board. We hope that the selected 15 manuscripts represent the current success and challenges of medical genomics research and indicate the future direction.

The genetic heterogeneity and the complicated molecular mechanism underlying tumor progression have made it highly difficult to develop sustainable approaches for cancer therapy. The explosion of knowledge about global gene alterations and genomic variations has changed the traditional approaches of cancer research and presented a promising foundation for diagnosis and treatment of cancer. Much effort of this supplement is devoted to cancer research with various types of genomics data in terms of early diagnosis, biomarker identification, cancer classification and therapy. Wang *et al. *[1] proposed a Chi-square statistic based Top Score Genes (Chi-TSG) method for tumor classification using global gene expression data. They tested multiple cancer datasets and found their algorithm had better performance in comparison to other widely-used classifiers, such as Top Scoring Pair, Support Vector Machines and Prediction Analysis of Microarrays. A manuscript by Zhang and Chen [2] also explored cancer classification by developing multiple distance metrics for proteomics data. Their data showed correct identification of breast cancer subtypes and molecular pathways. A predictive model, Support Vector Machine based on Recursive Feature Elimination and Cross Validation (SVM-RFE-CV), was proposed by Zhang, Deng and Drabier [3] for breast cancer early detection using microarray data collected from peripheral blood. Their method identified a small group of biomarker genes for early diagnosis, which is potentially more convenient and accurate than the traditional diagnosis using tissue biopsies and mammography. Cancer detection and prediction was developed in non-small cell lung carcinoma (NSCLC) by Tran [4]. His method based on generalized Lorenz curves and Gini ratios identified a set of 9 genes from the NSCLC gene expression profiles and these biomarker genes can be used for cancer prediction. Epigenetic variation can be used for cancer early detection and classification as well. Chen *et al. *[5] proposed a nonparametric method for detecting differentially methylated loci and suggested its application in cancer diagnosis and classification.

Gene pathway and networking is another major focus of this special issue. Garrett *et al. *[6] investigated the molecular changes for human proximal tubule cells in the short and long term exposure of cadmium. The short and long term exposure in cells represented the acute and chronic toxic response of human kidney. Varying exposure time and cadmium concentration enabled them to conduct gene pathway analysis and construct gene networks model for toxic response by heavy metal. Pathway analysis was carried out by Liu *et al. *[7] to investigate potential common molecular mechanism underlying two complex diseases, Schizophrenia and type 2 diabetes mellitus. The protein-protein network revealed high connectivity hub proteins that were involved in a few important signal pathways for both diseases. Gene order is an emerging way for gene clustering and molecular pattern discovery. By combining transcriptome data with lipidomics data, Zhao et al. [8] identified activated pathways during type 2 diabetes development. Hu *et al. *[9] compared a few distance metrics using genetic algorithm and Ant Colony Optimization for computing gene order and found that the optimized method worked well for Alzheimer's disease microarray data.

The manuscripts of this supplement cover various data types and broad topics. ChIP-Seq of RNA Pol II was used to identify the bidirectional promoter by detecting bi-peak shapes using computational approach [10]. Qureshi and Sacan [11] proposed a weighted normalization method for microRNA RT-PCR data. Juan *et al. *[12] investigated the interaction between microRNAs and long intergenic noncoding RNAs microRNAs. Such interactions were implied in breast cancer development. Wang *et al. *[13] developed a single gene sequencing technique for genotyping hepatitis B virus and detecting gene mutation for drug resistance. Zheng *et al. *[14] proposed a support vector machine-based machine learning method to predict DNA methylation status, which may have broad applications in disease studies. Teng, Yang and Wang [15] developed a machine learning method to predict the human tissue-specific genes from microarray data and UniProt database. Their method may be useful for understanding the molecular mechanisms underlying tissue-specific diseases. Lastly, we would like to introduce a database for disease associated mutations. The database, HDAM, is constructed by Jia *et al. *[16] using next generation sequencing data. HDAM currently assembled 1,114 mutations accounting for 669 genes and 125 human diseases.

In short, this special issue brings forth many exciting scientific discoveries and methodology advances that present the cutting edge development of genomics research in human diseases. We hope that it would provide guidance for the questions and attempts in current genomics research and stimulate ideas for future studies.

## Competing interests

The authors declare that they have no competing interests.
